# CMV-specific T-Cells for Treatment of CMV Infection after Hematopoietic Stem Cell Transplantation in a Pediatric Case: First Application in Turkey

**DOI:** 10.4274/tjh.galenos.2019.2019.0293

**Published:** 2020-02-20

**Authors:** Sevil Celilova, Ersin Toret, Başak Aksoy Adaklı, Ercüment Ovalı, Ceyhun Bozkurt

**Affiliations:** 1 Altınbaş University Faculty of Medicine, Medicalpark Bahçelievler Hospital, Department of Pediatric Hematology-Oncology & Bone Marrow Transplantation Unit, İstanbul, Turkey; 2 Acıbadem University Faculty of Medicine, Altunizade Hospital, Department of Hematology, İstanbul, Turkey; 3 İstinye University Faculty of Medicine, Medicalpark Bahcelievler Hospital, Department of Pediatric Hematology-Oncology & Bone Marrow Transplantation Unit, İstanbul, Turkey

**Keywords:** Childhood, Hematopoietic stem cell transplant, CMV, Specific T-cell, Therapy

## To the Editor,

Cytomegalovirus (CMV) infection is still a major complication after allogeneic hematopoietic stem cell transplantation (HSCT) [1,2]. Unfortunately, prolonged antiviral treatment of CMV infection causes a delayed CMV-specific immune reconstitution. At this point, adoptive immunotherapy by CMV-specific T-cells can control CMV infection or provide immune reconstruction [3,4,5].

**Figure 1:**
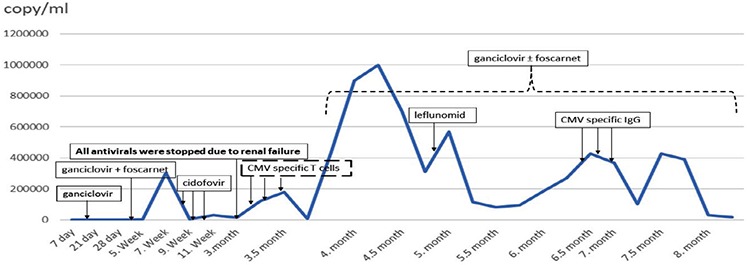
Treatment process according to the course of CMV DNA titer (copies/mL). CMV: Cytomegalovirus.

A 17-year-old boy with high-risk T-cell acute lymphoblastic leukemia underwent HSCT from one antigen-mismatched unrelated donor. He was conditioned with treosulfan, fludarabine, thiotepa, and rabbit anti-thymocyte globulin at 15 g/m^2^ for 3 consecutive days (days -2 to 0). The patient also received cyclosporine A (CsA) divided into two doses: 3 mg/kg daily from day -1 to post-transplant days +20 and +30 intravenously then switched to approximately 6 mg/kg peroral daily (targeted blood concentration: 200-250 ng/mL with monitoring). CsA was tapered quickly and stopped in the third month of transplant due to renal failure. Methotrexate was administered on days +1 (10 mg/m^2^), +3 (8 mg/m^2^), and +6 (8 mg/m^2^). He achieved neutrophil engraftment on day +17 and thrombocyte engraftment on day +32. Full donor chimerism was observed in the first and third months. Lymphoid engraftment was achieved on day +75 but generally the absolute lymphocyte count was under 1500/mm^3^. He was CMV immunoglobulin G (IgG)-seropositive and CMV-DNA polymerase chain reaction (PCR) was negative before transplantation. Unfortunately, his donor was CMV IgG-seronegative. CMV infection (reactivation) occurred on day +19. Ganciclovir was started at 10 mg/kg/day and no response was obtained in 14 days. CMV drug resistance mutation was detected in the *UL54* polymerase gene. Foscarnet was administered at 180 mg/kg/day on day +34. First, an increase of CD3+ lymphocytes was seen in the lymphocyte subtype analyses around the third month after the transplant. As a comorbidity, in spite of the fact that fluoroquinolone was administered until +30 day, BK virus infection developed in the patient and cidofovir was used at 5 mg/kg/week on days +52, +67, and +79. No response was achieved with the antiviral treatment and renal failure developed in the patient on day +82. All antivirals were stopped. According to the recent literature, the transplant council decided to use CMV-specific T-cells for the patient’s ongoing CMV infection. Informed consent was received from his family and the application was approved by the Ministry of Health’s Scientific Advisory Commission on Stem Cell Transplantation. In accordance with cGMP standards, peptide-specific T lymphocytes were isolated and amplified by a interferon-γ cytokine capture system using the fully automated CliniMACS Prodigy device at Acıbadem Labcell, İstanbul. The infusion doses of third-party CMV-specific T-cells were 2x10^4 ^cell/kg and 1x10^4 ^cell/kg in the 20^th ^and 22^nd ^weeks after transplantation, respectively. While the recommended dose of T-cells was 2x10^6^/m^2 ^[6], we reduced the dose due to the risk of graft-versus-host disease (GvHD). The CMV-DNA PCR level was higher than 1x10^5 ^copies/mL before infusion and had decreased to 8x10^4 ^copies/mL on the 15^th^ day after infusion. The patient had no immunosuppression at the time of T-cell infusion and did not develop GvHD after the infusion. In follow-up, CMV-DNA PCR increased to more than 3.5x10^5 ^copies/mL in the first month of the cell infusion and the sixth month after transplantation. In this period, CD3-CD16+56+ (natural killer) and CD3+CD8+ (T cytotoxic) lymphocyte subtypes were increased. Nevertheless, the patient developed respiratory distress and CMV infection was detected from the bronchoalveolar lavage sample, and the CMV DNA titer was 152,000 copies/mL. After losing partial response to CMV-specific T-cells, CMV pneumonia was proved and then leflunomide was tested, but there was no response. Finally, CMV-specific IgG was administered once weekly three times. This treatment managed to decrease the CMV DNA copies to under 20,000 copies/mL. The treatment process according to the course of CMV DNA titer is shown in Figure 1.

CMV reactivations/infections are life-threatening complications in the transplant setting, especially if the recipient and donor are CMV mismatches. From our experience with this case, CMV-specific T-cells can control viral replication to a certain extent, but not enough for permanent results. The answer may be CMV-specific IgG, which controlled CMV reactivation best in our case, and antivirals may be used in combination.
